# A 32-year species-specific live fuel moisture content dataset for southern California chaparral

**DOI:** 10.1038/s41597-026-06794-3

**Published:** 2026-02-12

**Authors:** Kevin Varga, Charles Jones

**Affiliations:** 1 Department of Geography, University of California Santa Barbara, Santa Barbara, USA; 2 Earth Research Institute, University of California Santa Barbara, Santa Barbara, USA; 3 School of Integrated Sciences, James Madison University, Harrisonburg, USA

**Keywords:** Fire ecology, Atmospheric dynamics, Natural hazards, Ecological modelling

## Abstract

Live fuel moisture content (LFMC) strongly affects the behavior of wildland fire, resulting in its incorporation into wildfire spread models and danger ratings. In this study, over ten thousand LFMC observations are combined with predictor variables from Landsat imagery and the Weather Research and Forecasting model to train species-specific random forest models that predict the LFMC of four fuel types—chamise, old growth chamise, black sage, and bigpod ceanothus. These models are then utilized to create a historical, 32-year long, LFMC dataset in southern California chaparral. Additionally, the high spatial and temporal sampling frequency of chamise allowed for quantile mapping bias correction to be applied. The final chamise output, which is the most robust, has a mean absolute error of 9.68% and an R^2^ value of 0.76. The LFMC dataset successfully captures the variability in the annual cycle, the spatial heterogeneity, and the interspecies differences, which makes it applicable for better understanding varying fire season characteristics and landscape level flammability.

## Background & Summary

As a wildland fire burns across a landscape, the behavior is often highly heterogenous, with stretches of scorched canopies intermixed with unburned islands. One of the many factors influencing this heterogeneity is live fuel moisture content (LFMC), calculated as the ratio of water to dry matter within vegetation. LFMC is typically expressed as a percentage. It can dip down to 30%, below which is considered dead fuel, or reach over 300%, with variations across different ecosystems, species, and even within individual plants^1^. LFMC is influenced by a myriad of factors, including atmospheric conditions^2–5^, plant physiology^6–11^, and soil characteristics^12,13^. Soil moisture fundamentally affects plant water potential, while plant hydraulic traits dictate how much water moves through the system. Additionally, water is crucial for photosynthesis, which in turn creates more plant matter^14^. Atmospheric conditions play a large role by affecting water availability, vapor pressure deficit, evapotranspiration, and solar radiation^15^. By understanding how these variables affect LFMC, it is then possible to model LFMC dynamics, which can further aid in understanding wildland fire behavior.

Fire behavior starts at the combustion level, which is directly affected by LFMC. If enough heat is applied to a solid fuel, the molecules break down into gases, a process known as pyrolysis. Continued heating then causes combustion of those gases^1,16^. The reaction rate of pyrolysis and combustion escalates exponentially with temperature, and greater rates of combustion increase a fire’s heat release rate, which drives wildfire spread. In order for combustion to occur, water must be driven off, with the high latent heat of vaporization acting as a thermal sink, slowing these processes. The release of water vapor also dilutes the pyrolyzed combustible gases, thereby reducing combustion efficiency^16^. A flammability study of California chaparral species quantified LFMC-driven combustion delays by calculating an empirical relationship between the moment of full combustion and maximum flame height, where every increase of 10% LFMC caused an additional four second delay^17^.

Due to its importance, LFMC has long been considered in fire danger rating systems, including the United States National Fire Danger Rating System (NFDRS)^18^. Initially, LFMC was simply modeled as very wet dead fuels, disregarding the dynamic nature of plant physiology where moisture and carbon are constantly in flux^6^. Currently, LFMC within the NFDRS is modeled with the Growing Season Index, which aims to predict ecophysiological limits of photosynthesis by combining the limiting effects of temperature, water availability, and day length^18^. Other studies have used meteorological variables or drought indices to predict LFMC with some success^4,19^, but these methods do not adequately represent vegetation conditions or soil moisture. Furthermore, remote sensing-based LFMC modeling methods are able to monitor vegetation across vast regions^20–22^, but problems persist, including heterogeneity within pixels, differing relationships between spectral indices and vegetation types, and varying instrumentation spatiotemporal resolution and lifespan^23^. More recently, mechanistic models of LFMC have been developed that use physiological metrics for plant water content, such as relative water content or leaf water potential, and for plant dry mass, such as leaf density or leaf mass area^9,11,24,25^. The separation of the LFMC components helps to understand species-specific drivers of LFMC variation.

Many wildfire prone areas across the United States, and some across the world, sample LFMC, but the locations are sporadic and often only focus on one species^26^. As mentioned before, wildfires, and LFMC, can be heterogenous across a landscape, making spatially varying LFMC values important in wildfire behavior models, both operationally and in hindcast modeling analyses^27–31^. McCandless *et al*.^21^, sought to better inform real-time wildland fire management by using remote sensing, soil moisture, and surface weather variables to model daily LFMC and dead fuel moisture across the Conterminous United States beginning in 2015. While the methodology is sound, the LFMC predictions are species-agnostic, which does not represent the large interspecies variations and potentially caused larger errors. Additionally, the satellite that was used aged out, causing an end to data production^21^. Similarly, another promising species-agnostic gridded LFMC product, created by Rao *et al*.^20^, only produced data between 2016 and 2021, due to failure of the Sentinel-1B satellite. There are still many unanswered questions about how LFMC affects fire spread in different fuels, as well as how different species’ LFMC responds to varying atmospheric conditions. For this reason, we set off to create a species-specific, long-term LFMC gridded dataset that could also be applied to other species in other regions.

**Fig. 1:**
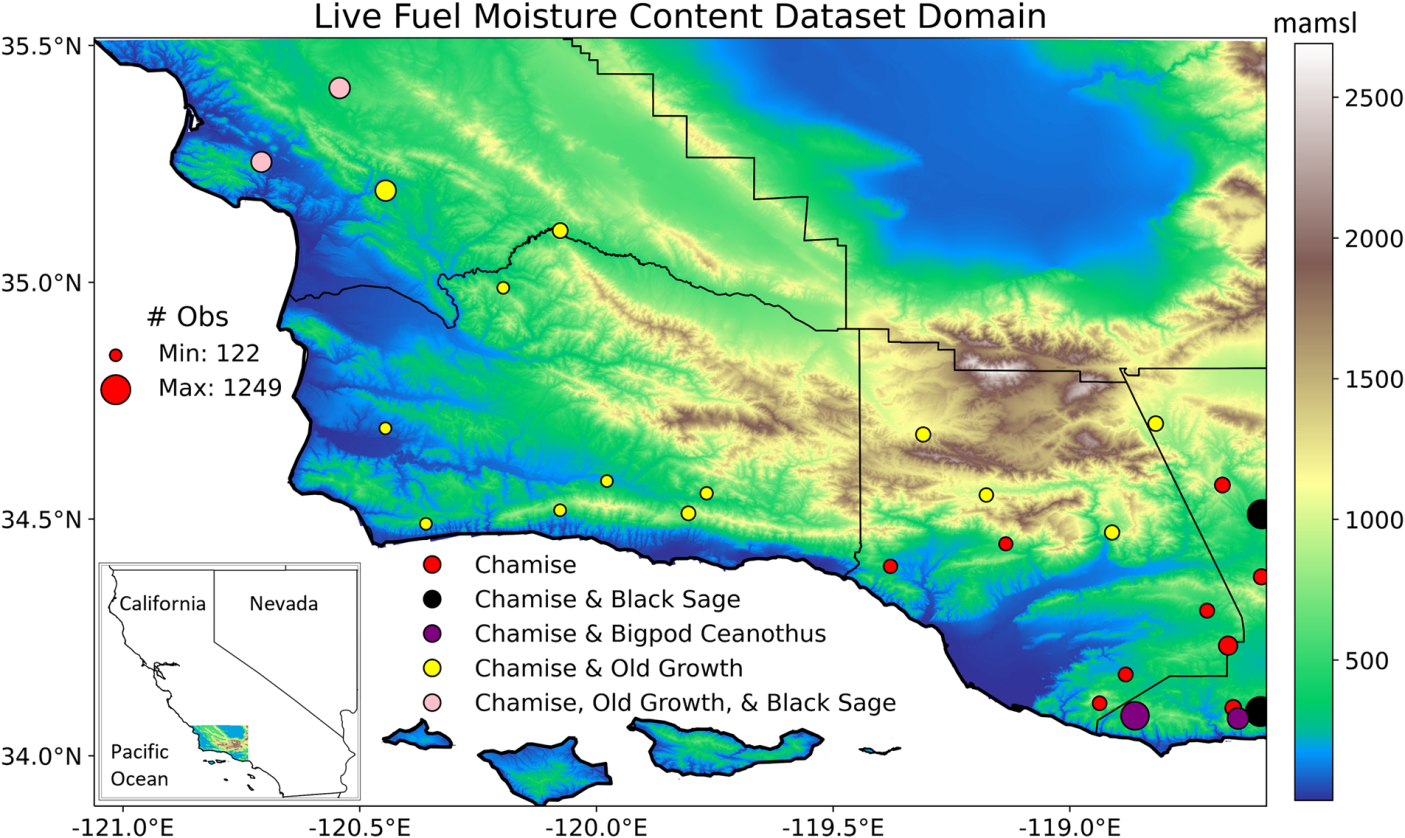
Elevation of spatial domain, which stretches from sea level to over 2500 meters above mean sea level (mamsl), with LFMC observation sites shown, differentiated by the number of observations and the fuel types that were sampled.

In this study, we combine numerical weather modeling and remote sensing data to create predictive random forest models that output 32-year (Dec 1987 – Jun 2019) historical LFMC datasets of four shrubland fuel types: new growth chamise (*Adenostoma fasciculatum*), old growth chamise, new growth black sage (*Salvia mellifera*), and new growth bigpod ceanothus (*Ceanothus megacarpus*). New growth refers to the foliage and terminal stems of the current growing season, while old growth is foliage and terminal stems from past growing seasons^32^. The spatial domain stretches from San Luis Obispo County to the edge of Los Angeles County, in California (Fig. 1), aligning with a 1 km climatology created with the Weather Research and Forecasting (WRF) model^33^, which is used for model predictors. Imagery from NASA’s Landsat mission is also used for a model predictor^34^.

This California coastal mountain area has a Mediterranean climate with a pronounced summer dry season. Shrublands, specifically the highly flammable California chaparral ecosystem, are the dominant vegetation type, covering 35% of the land area^35^. Chamise shrubs often comprise 50–90% of chaparral stands, leading to fire agencies using chamise LFMC as a fire danger indicator. Black sage commonly contributes 20–50% of shrub canopy cover, while bigpod ceanothus is often 10–30% of cover^36^. LFMC measurements of these species have been recorded throughout this area and time period, mostly by fire agencies to asses fire danger. Over 10,000 LFMC observations were obtained from the US National Fuel Moisture Database^37^ and Santa Barbara County Fire Department^38^ for model training and testing. The fuel specific models output LFMC values on the first and the fifteenth of each month, following the typical sampling schedule of fire agencies. The long data records of the products, especially the bias-corrected chamise dataset, provide an important resource to understand the spatiotemporal variability of LFMC and fire behavior in southern California chaparral.

## Methods

**Fig. 2:**
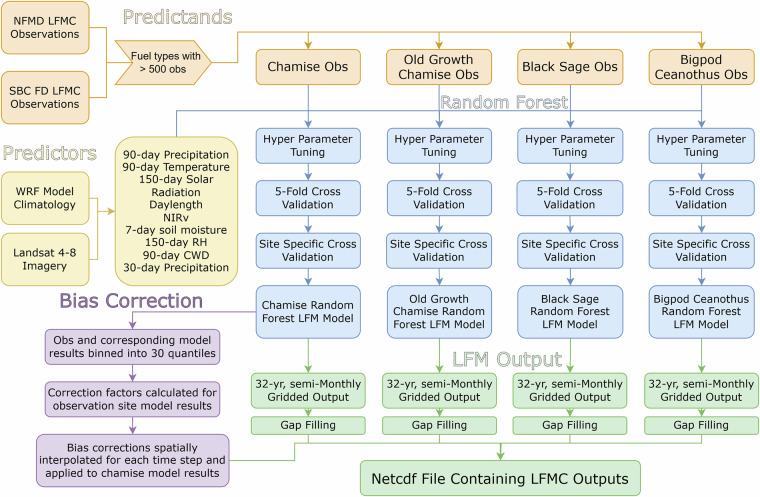
Flowchart depicting the process of dataset creation.

The following sections will describe dataset development, from initial predictor testing to cross validation checks. To start, LFMC observations were acquired as the model predictands. Then, predictors were calculated, including long-term lag variables, such as 90-day precipitation, 90-day mean temperature, and 150-day mean insolation, as well as short-term lag or instantaneous predictors, such as day length, 7-day mean soil moisture, and near-infrared reflectance of vegetation (NIRv). Species-specific random forest models were then trained and tested with two techniques: 5-fold cross validation using all observations and observation site specific cross validation, where each site was individually withheld as the test data. After testing, models were trained on all predictors/predictands and used to create the species-specific LFMC datasets. Lastly, quantile mapping bias correction was applied to the modeled chamise LFMC dataset, which was possible due to the high spatial frequency of observation sites. A visual flowchart summary of the dataset creation is shown in Fig. 2.

### LFMC observation predictands

All LFMC observation sites within the spatiotemporal domain were identified (Fig. 1). All fuel types with more than 500 observations were used, which included chamise, old growth chamise, black sage, and bigpod ceanothus, with chamise being by far the most numerous (Table 1). Black sage (4) and bigpod ceanothus (2) were sampled at significantly fewer sites, but their observation records covered most of the temporal domain. On the other hand, old growth chamise has been sampled at more locations (15) but has a shorter observation record. Most of the observations were provided by the US National Fuel Moisture Database program via the pyNFMD repository on GitHub^37^. We also retrieved additional LFMC observations from Santa Barbara County Fire Department^38^, as they were not included in pyNFMD. Santa Barbara County Fire Department (FD), Ventura County FD, Los Angeles County FD, Kern County FD, CAL FIRE San Luis Obispo Unit, and the Los Padres National Forest were the agencies that collected samples. LFMC sampling protocols are based on the US Forest Service Fuel Moisture Collection Methods. According to the Collection Methods, new growth is typically sampled, unless otherwise specified, such as with chamise old growth. New growth consists of the current growing season’s fresh foliage and stems, while old growth consists of any past foliage and stems^32^.

**Table 1:** The modeled fuels, number of live fuel moisture content observations, number of sampling sites, and temporal domains for each fuel type.

Fuel Type	# Obs	# Sites	First Obs	Last Obs
Chamise	6404	28	1/12/1990	6/29/2019
Old Growth Chamise	1426	15	5/1/2006	6/29/2019
Black Sage	1247	4	1/12/1990	6/28/2019
Bigpod Ceanothus	580	2	1/12/1990	11/13/2018

Sampling sites had observations labeled chamise, chamise new growth, and chamise old growth. After comparing distributions of the three, the chamise labeled observations corresponded to typical chamise new growth values, so chamise new growth labels were converted to just chamise. When sites had two observations of the same fuel type on the same day, the mean was used. One sampling site, Frazier Park, was removed due to poor data quality, evident when comparing the values to spatially proximal sites. Another site was removed due to it having less than 25 observations.

### WRF predictors

For every observation, predictor variables were calculated from a previously constructed WRF model, (Advanced Research WRF version 4.0.1) 32-year (1987–2019) high-resolution downscaling climatology^33^, available via the CLIVAC lab at UC Santa Barbara^39^. The WRF downscaling dataset utilized 4 two-way nested grids (27 km, 9 km, 3 km, 1 km) and numerical parameterizations for microphysics (Single moment 6-class)^40^, long-wave and short-wave radiation transfer (RRTMG)^41^, Noah multi-parameterization options (Noah-MP)^42^, surface layer physics and planetary boundary layer (MYNN)^43,44^. The choice of parameterizations, as well as 1 km resolution inner domain, have proven to produce accurate surface weather variables in other studies that investigate fire danger in association with downslope Sundowner winds in the area^45–48^. European Center for Medium-Range Weather Forecasts Interim Reanalysis (ERA Interim) was used for initial and boundary conditions, with updates on sea surface temperature every 6 hours. The 27 km outer WRF domain included grid nudging, which forces WRF to stay aligned with the reanalysis data. For predictor variable pre-processing, WRF hourly outputs from the 1 km domain were processed into daily accumulated precipitation and mean daily outputs of temperature, relative humidity, incoming shortwave radiation, soil moisture, actual evapotranspiration (AET), surface pressure, net radiation, saturation vapor pressure, actual vapor pressure, and wind speed.

Vegetation physiological dynamics are affected by preceding meteorological conditions. As such, we tested a number of short term and long-term lag variables from WRF, inspired by a previous study^19^ (Table 2). Testing involved comparing the influence each predictor had on model accuracy by calculating Gini importance and cross validation statistics. All WRF predictors are direct outputs of the model, except reference evapotranspiration (ETo), which was calculated with the Penman-Monteith equation using WRF outputs^49^. WRF modeled AET is then subtracted from ETo to get climatic water deficit, which has been shown to affect LFMC^4^. We mostly utilized direct outputs from WRF, rather than composite drought indices, to enable direct attribution of LFMC response to individual meteorological drivers via Gini importance. Drought indices have previously been used to estimate vegetation moisture content, but they are an imperfect method^50^.

**Table 2:** Initial and final model predictors.

Initial Tested Predictors		Final Predictors
Day Length	90-day total precip	90-day total precip
3-day max temp mean	90-day mean temp	90-day mean temp
7-day max temp mean	150-day mean temp	150-day solar radiation
7-day max RH mean	150-day mean RH	NIRv
3-day total precip	150-day solar radiation	Day Length
7-day total precip	Elevation	7-day mean soil moisture
7-day mean soil moisture	Slope	150-day mean RH
30-day wind speed	Aspect	90-day climatic water deficit
30-day mean VPD	NDVI	30-day total precip
30-day total precip	NDMI	
90-day ref evapotranspiration	NIRv	
90-day climatic water deficit		

For investigating predictor variable lag times, we conducted an autocorrelation analysis for each initial tested predictor (Table 2), where LFMC observations, as well as each potential predictor, were resampled to a semi-monthly temporal frequency. Precipitation, ETo, and climatic water deficit were summed over the semi-monthly periods, while all other variables were averaged. The highest correlation lag time was averaged across each LFMC observation site for each variable. For example, when averaging all sites, accumulated precipitation showed the greatest correlation after 3.9 periods, or about 58 days, so 58-day accumulated precipitation was tested as a predictor. Interestingly, after model testing, the initial lag times that were used, mainly 90- and 150-day, performed better than the computed lag times, resulting in the use of the initial lag times for the final predictors.

**Fig. 3:**
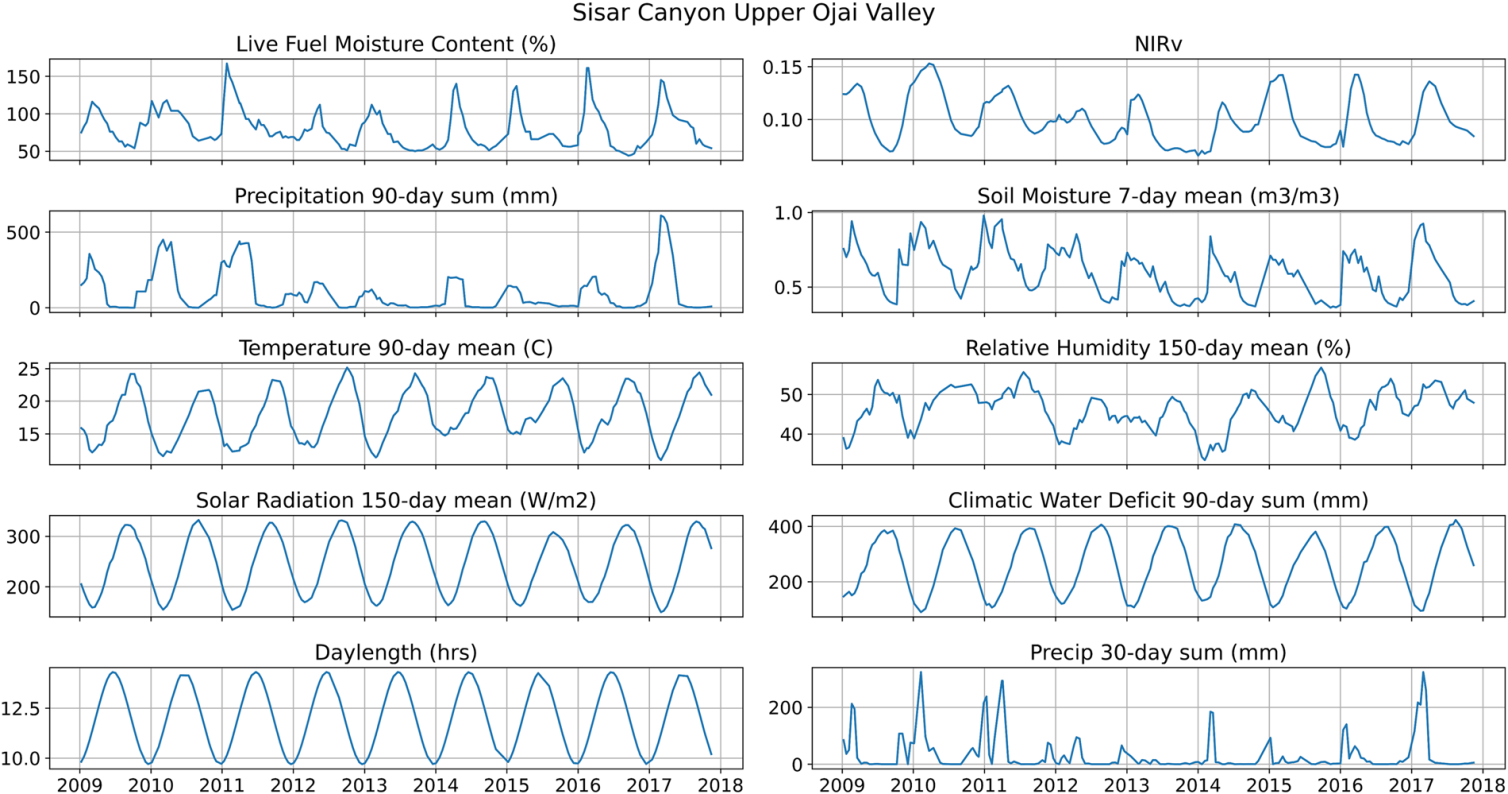
Live fuel moisture content observations at one sampling site, Sisar Canyon Upper Ojai Valley, plotted with the nine model predictors.

After final predictor selection, daily values for each predictor were calculated across the entire domain. Due to seasonal cycles, many of the predictors are correlated with each other (Fig. 3). However, random forests are robust to multicollinearity because predictors are randomly selected at each split within each decision tree, preventing correlated predictor pairs from dominating the ensemble. Bootstrapped sampling further reduces any negative effects of collinearity by randomly selecting a subset of predictors to use in each decision tree^51^. The WRF downscaling dataset extends from June 1987 through June 2019, but due to the 150-day lag variables, our predictors, and thus final LFMC dataset, stretch from December 1987 through June 2019.

### Landsat predictor

To incorporate vegetation conditions, we calculated vegetation indices from Landsat 4–8 imagery^34^, due to the historical temporal coverage and expected future operational availability. Initially, the normalized difference vegetation index (NDVI), normalized difference moisture index (NDMI), and near-infrared reflectance of vegetation (NIRv) were all tested as predictors. Each of these three vegetation indices influenced the model in similar ways, with NIRv increasing model accuracy the most. Therefore, NDVI and NDMI were dropped (Table 2). NIRv is calculated by multiplying near-infrared reflectance (NIR) of a scene by NDVI. NIRv was first introduced to more accurately calculate terrestrial photosynthesis, partially by alleviating the mixed-pixel problem—remote sensing pixels including non-vegetated surfaces^52^. The mixed-pixel problem is important in our domain, which includes urban areas, bare ground, and agriculture. Due to vegetation having high reflectance of NIR, multiplying NIR by NDVI effectively pulls out the vegetation signal. We calculated semi-monthly NIRv values across our spatiotemporal domain using a previously published Google Earth Engine (GEE) based algorithm^53^.

The algorithm goes as follows. First, Landsat images within individual years, and within our spatial domain, are passed through the GEE provided cloud filter before calculating NIRv. Another cloud filter focuses on cloud shadows by removing values that substantially deviate below a linear regression of the neighboring NIRv values. Semi-monthly values are then calculated by looking at all values within a 120-day window, centered on the desired time step and overlapping preceding and proceeding years. If there are more than five Landsat images in that window, a quadratic regression is used to estimate the NIRv value at the desired time step. If there are between three and five images, a linear regression is used. If there are less than three values, the median value is used. These NIRv values are then upscaled from 30 m to 1 km resolution and exported from GEE. The NIRv geoTIFF files are converted to the WRF latitude/longitude grid, masked with a land mask, and cropped to the temporal domain of the WRF predictor variables. NIRv values below zero were removed, as these values represent water, and one outlier above 0.5 was removed. For the final step, temporal linear interpolation was used to fill any NIRv data gaps less than 90 days.

### Random forest model development

With predictor variables finalized, values were linearly interpolated from their gridded output to the location and date of each LFMC observation. The predictors were then normalized by subtracting the mean and dividing by the standard deviation, which was done for every model iteration. Random forest models were chosen as the predictive model, due to the efficient computing, interpretability, and LFMC prediction success in other studies^19–21,54^. Random forest hyper parameter tuning was conducted by creating a parameter grid of bootstrap use setting, maximum depth, maximum features, minimum samples per leaf, minimum samples per split, and number of trees. For each of the four fuel types, 5-fold cross validation was performed on 100 different randomized iterations of the parameter grid. The parameter settings that resulted in the lowest R^2^ value for the test sets were saved as the tuned parameter combination for each fuel type and used for every model iteration (Table 3).

**Table 3:** Hyper parameters for random forest models determined via randomized grid search.

Fuel Type	# Trees	Min Samples Split	Min Samples Leaf	Max Depth	Max Features	Bootstrap
Chamise	800	2	2	50	3	FALSE
Old Growth Chamise	1000	2	1	20	3	TRUE
Black Sage	1000	2	1	110	3	TRUE
Bigpod Ceanothus	1000	2	1	20	3	TRUE

Using all available predictors—normalized as described above—and predictands for training, fuel specific random forest models were fit using the derived hyper parameter settings. The models were then used to calculate fuel specific LFMC values at 1 km spatial resolution on the first and the fifteenth of every month from December 1987 through June 2019. Many of the time steps in the modeled LFMC data included small spatial gaps, especially along the coast. These spatial gaps are attributable to constant cloud cover occurring during Landsat passes, which results in missing NIRv values. All of the LFMC spatial gaps were identified for every time step and determined to be small enough to be fillable via spatial linear interpolation, providing dataset consistency across space and time.

### Chamise quantile mapping bias correction

When comparing the modeled chamise LFMC against the observations, the modeled results at some observation sites exhibited consistent biases, especially during the peak periods of the wet and dry seasons. We determined that quantile mapping bias correction could be applied to the chamise LFMC dataset, due to the high spatial coverage of observation sites (Fig. 1). To do so, chamise LFMC observations were resampled to the semi-monthly temporal resolution of the modeled results, allowing for quadratic interpolation of two time steps. In order to optimize the bias corrected results, different numbers of quantiles were tested: 10, 20, 30…100, with 30 quantiles causing the greatest reduction in mean bias error across the sites. As such, 30 quantile values were calculated for the LFMC observations and their corresponding model predictions at each site. The differences between the quantile values were calculated in order to determine the typical bias correction that was needed for the range of modeled values. Modeled values over the full temporal domain were then fit into the aforementioned model quantile bins, with the corresponding correction factor then applied to each bin.

**Fig. 4:**
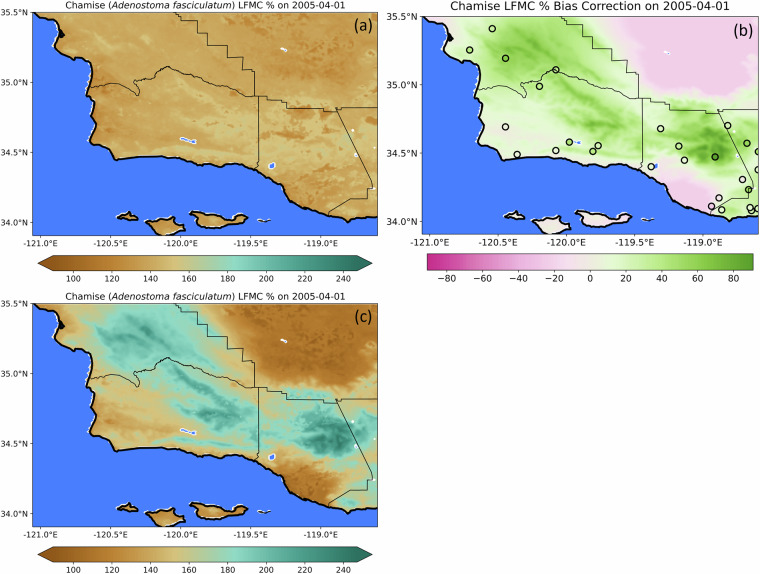
Initial modeled chamise live fuel moisture content (**a**), bias correction applied (**b**), and bias corrected chamise LFMC (**c**) at the time step with the greatest bias correction value.

With temporally varying bias correction factors calculated at all 28 chamise LFMC observation sites, spatial interpolation of the corrections at every time step was performed using latitude, longitude, and elevation, across the 1 km resolution grid of modeled chamise LFMC values. A thin plate spline driven radial basis function was used, due to the efficient implementation across multiple dimensions. Some sites are close in proximity, which makes it difficult for the interpolation to exactly fit the input data. Therefore, a smoothing factor of 0.01 was used to allow for the fitting of the interpolated corrections to be based on a least-squares fit. Once gridded bias correction factors were calculated for every time step, these were added to the original modeled chamise LFMC values to produce a bias corrected dataset. Figure 4 visualizes the bias correction of the time step with the greatest correction value.

## Data Records

The modeled LFMC of four fuel types—new growth chamise (*Adenostoma fasciculatum*), old growth chamise, new growth black sage (*Salvia mellifera*), and new growth bigpod ceanothus (*Ceanothus megacarpus*)—and the bias corrected chamise LFMC are available as a single NetCDF file on Dryad, with 1 km, semi-monthly spatiotemporal resolution and latitude, longitude, and time (December 1987 – June 2019) as the dimensions^55^. There are LFMC values for every land grid point in the domain, even though these fuel types do not occur in many locations. The chamise dataset, especially the bias corrected chamise dataset, is considered to be the most robust, due to fire agencies preferentially sampling chamise in this domain, as well as the species prevalence in chaparral ecosystems^56^ (Fig. 1, Table 1).

## Technical Validation

**Fig. 5:**
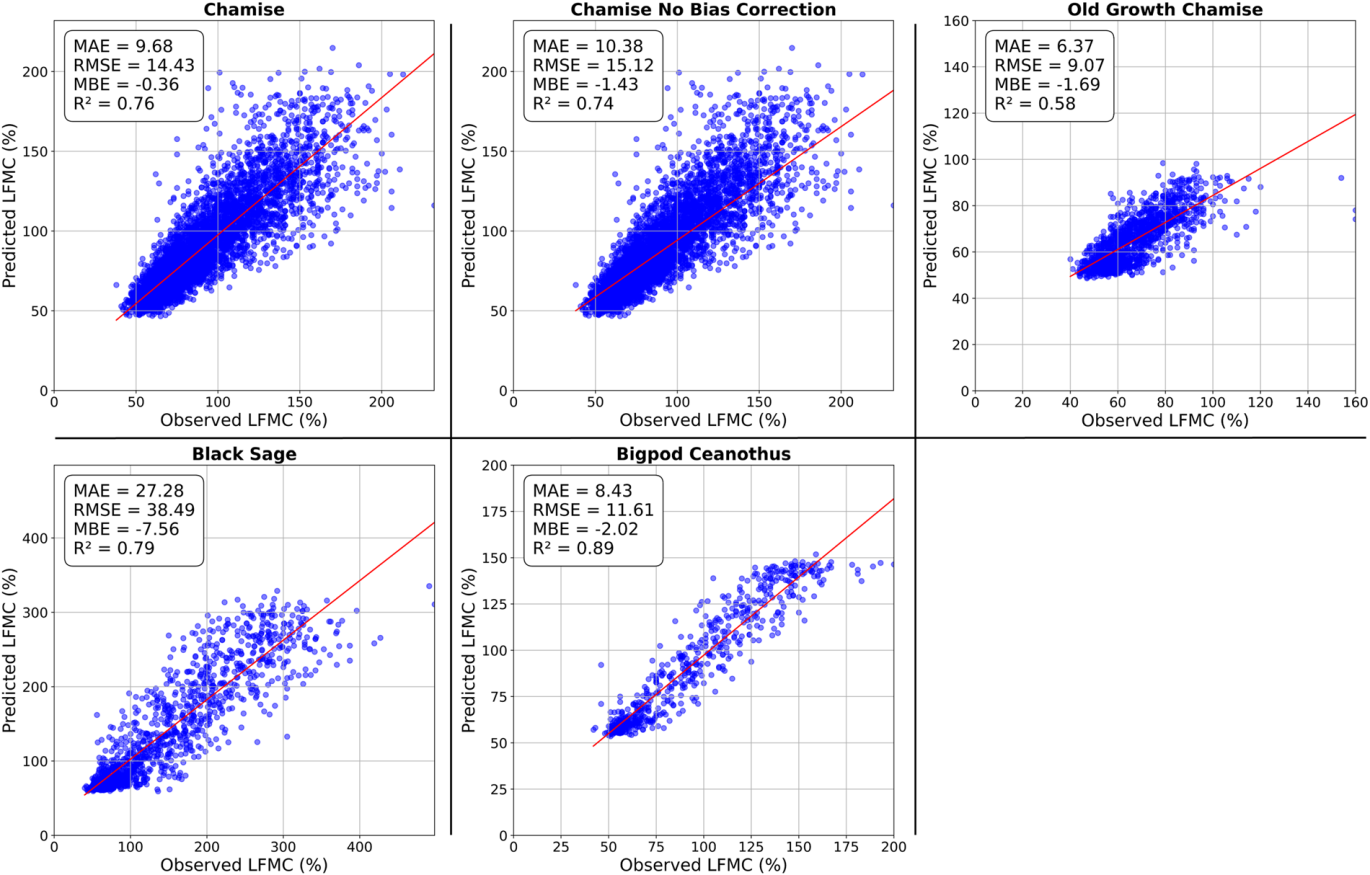
The observations for each fuel type are plotted against modeled values that are linearly interpolated to the time and location of each observation. Mean absolute error (MAE), root mean square error (RMSE), mean bias error (MBE), and R^2^ are shown for each model. The red line indicates the ordinary least squares regression.

For final validation, the modeled outputs were linearly interpolated, both spatially and temporally, to the time and location of every LFMC observation of the respective fuel types. Figure 5 shows scatter plots between the observations and predictions, with associated statistics. The models all tend to over predict at low observational values and under predict at high observational values. This tendency is common in random forest models, due to the averaging effect across many decision trees^57^. Prediction accuracy at the lower end of LFMC is more important for fire danger prediction. Specifically for chamise, a past study determined that large fire growth is more common below a 79% LFMC threshold^3^. For our bias corrected chamise model predictions of observed LFMC below 79%, the MAE is 5.9%, the MBE is 1.47%, and the RMSE is 8.22%. The positive MBE in this lower range and the overall negative MBE of all models shows that the bias on the higher end of predicted values is more pronounced.

Model prediction uncertainty is also quantified by using the variance among all the decision trees employed for each individual prediction. Specifically, the standard deviation is computed using the range of predictions from every tree. A 95% prediction interval is then calculated as the mean prediction ±1.96 standard deviation. This approach leverages the natural ensemble spread of the forest to estimate local predictive uncertainty. The method follows the quantile regression forest framework, where tree variance reflects epistemic uncertainty due to model structure and training data sampling^58^. Uncertainty fields are provided in the dataset as separate variables with the same spatiotemporal dimensions as the LFM predictions.

## Usage Notes

### Limitations

The dataset presented in this study comes with a few limitations. First, LFMC sampling locations are spatially sparse, especially for black sage and bigpod ceanothus (Fig. 1). Therefore, the potential spatial heterogeneity of intraspecies LFMC behavior may not be captured during model training. Old growth chamise has greater spatial coverage, but the observational record was temporally shorter. Due to these limitations, the chamise dataset is considered the most robust, although 28 sampling sites is still not widespread spatial representation. Second, the datasets provide LFMC values for every land grid point, even though any particular fuel type does not occur in many areas. It is recommended that land cover data is used to mask the LFMC data in order to focus on regions where the results are more appropriate, such as shrublands. Specifically, modeled LFMC results in agricultural or developed areas may not be accurate, due to differences in NIRv values between native shrubs and irrigated crops or vegetation.

Additionally, the construction of this dataset was based on the spatiotemporal domain and data availability of a previously constructed, 32-year downscaled climatology. While this type of historical, downscaled data is not available everywhere, the predictor variables can also be calculated using other climatological datasets. We specifically sought to combine meteorological predictors, which represent drivers of vegetation green-up and senescence, and current condition predictors—NIRv, day length, soil moisture—that would allow for this LFMC predictive methodology to be applied to other fuel types in other regions. It is the authors’ intent to expand this historical dataset to the entire state of California.

### Application

The potential usage of this dataset spans different disciplines and sectors. For example, fire agencies consistently sample LFMC to determine current fire danger. In the Mediterranean climate of California, the wet-up and dry-down behavior of LFMC determine when fire season starts and ends in different areas. With a historical LFMC dataset, additional analyses can lead to a better understanding of how meteorological and climatological factors influence LFMC behavior on a year-to-year basis. Those factors can then be used to help predict and prepare for upcoming fire/dry seasons. Other widespread LFMC datasets do exist^20,21,59^, but the combination of locally produced climatological data, the long Landsat lifespan, and the species-specific outputs, make this LFMC dataset valuable for local studies of fire dynamics.

The authors of this paper also contribute to a holistically-minded Regional Wildfire Mitigation Program (RWMP), which promotes living with fire instead of fighting it^60^. The landscape level management vital to creating fire resilient communities requires an understanding of the varying flammability of the surrounding natural fuel types. Historically, an emphasis has been placed on understanding when chamise LFMC reaches critical flammability thresholds^3,4^, but it is also important to understand how other species’ LFMC varies in space and time. For example, Figure 8 indicates chamise has the shortest wet season, when comparing fuel types on the same LFMC scale. Could other, less-flammable species be promoted around communities to decrease the fire risk? And how will flammability change as chaparral species distribution evolves with climate change? These are questions that a historical LFMC dataset can help answer.

## Data Availability

The LFMC dataset described in this work is available on Dryad (10.5061/dryad.rjdfn2zkw). The LFMC observations used for training the models are also available on Dryad. The observations were downloaded from the US National Fuel Moisture Database (https://github.com/wmjolly/pyNFMD) and the Santa Barbara County Fire Department (https://sbcfire.com/wildfire-predictive-services/). The data used for LFMC model predictors is publicly available via the UCSB CLIVAC Lab (https://clivac.eri.ucsb.edu/clivac/SBCWRFD/index.html) and the NASA Landsat program (10.1016/j.srs.2023.100103).

## Code availability

The codebase for the creation of this dataset is publicly available as Jupyter Notebooks on GitHub (https://github.com/kcvarga7/sba_lfm_1987-2019). All code was written in Python, with the exception of the GEE script used for downloading Landsat imagery. That GEE script is referenced in the GitHub readme, as well as the applicable Jupyter Notebook.
